# Strongly solved Ostle: calculating a strong solution helps compose high-quality puzzles for recent games

**DOI:** 10.7717/peerj-cs.1560

**Published:** 2023-09-12

**Authors:** Hiroki Takizawa

**Affiliations:** 1 Graduate School of Frontier Sciences, University of Tokyo, Kashiwa City, Chiba Prefecture, Japan; 2 Current Affiliation: Preferred Networks, Inc., Chiyoda-ku, Tokyo, Japan

**Keywords:** Retrograde analysis, Solving games, Ostle, Succinct data strucrture, Bitboard

## Abstract

Pure strategy board games such as chess are popular intellectual activities, and solving them is a challenging task in computer science. In addition to traditional games, many new board games have gained popularity in recent years. Ostle is one such unsolved game published in 2017. It is based on simple rules but is highly competitive. It is a two-player zero-sum game with perfect information in which the game-theoretical values of all game states can be obtained. In this study, we strongly solved Ostle by retrograde analysis. Utilizing various known techniques, including bitboards and succinct indexable dictionaries, significantly reduced the memory consumption in the analyses. We confirmed that the initial position is a draw and found some fundamental properties of Ostle. Additionally, we manually composed a tactical Ostle puzzle with the help of extracted outputs of the analyses. The result demonstrates that solving recent games provided helpful information to compose high-quality problems.

## Introduction

Computational solving of pure strategy board games such as chess, go, and checkers has been one of the goals of computer science. In the early days of computer science, Charles Babbage described the concept of automatically solving board games in his autobiography (Babbage, 1864). Various pure strategy board games are solved to date, and the most famous study is seemingly solving checkers (Schaeffer et al., 2007). In order to solve checkers, they used various algorithms: retrograde analysis (Thompson, 1986), alpha-beta search (Knuth & Moore, 1975) of superhuman-strength checker-program named Chinook (Schaeffer, 1997), and Df-pn (Nagai, 2002; Kishimoto et al., 2012). Df-pn is based on proof-number search (Allis, van der Meulen & van den Herik, 1994).

Solving games can be categorized as follows (Allis, 1994):

Ultra-weakly solved
: If the game-theoretic value of the initial position is determined, then the game is ultra-weakly solved. Note that this definition does not require any actual winning strategy.
Weakly solved
: If a strategy to achieve the game-theoretic value of the game for both players, from the initial position, under reasonable computational resources, then the game is weakly solved. For example, checkers was weakly solved in this sense (Schaeffer et al., 2007).
Strongly solved
: If the game-theoretic values of all possible legal positions are determined for both players, then the game is strongly solved. Note that a winning strategy can easily be obtained once a strong solution is given (*i.e.*, the theoretical values of the positions after each legal move from the current position can be seen). Retrograde analysis (Thompson, 1986) is a standard method for strongly solving a pure strategy board game.

Note that the term “games” here indicates only pure strategy board games. In contrast, when strongly solving imperfect-information games such as poker, the term “solving” basically means computing Nash equilibrium strategies for all possible positions. It is worth noting that the examination of imperfect-information games (Bowling et al., 2015) are out of scope of this study. Notably, some studies proposed even stronger categories than the above mentioned ones (Schaeffer & Lake, 1996; Gévay & Danner, 2015). They considered models in which opponent probabilistically makes mistakes, but those models fall outside the scope of this study.

Solving games is a different concept from developing superhuman-strength programs for games, as they do not necessarily require an analytic solution. Therefore, much research reported superhuman-strength programs for large-scale games that seem intractable to solve even ultra-weakly, such as go (Silver et al., 2016), chess (Campbell, Hoane & Hsu, 2002), and reversi (Buro, 1997). In contrast, developing a superhuman-strength program is straightforward once a game is weakly or strongly solved.

Several popular pure strategy board games have yet to be solved, and the number of such unresolved games continues to increase. In recent years, many new pure strategy board games have emerged, and some have become popular. Ostle, published in Japan in 2017, is one of them. In addition to games created by human game designers, some games generated by AIs have become popular, such as Yavalath (Browne & Maire, 2010; Browne, 2011).

In this study, we strongly solved the game Ostle by retrograde analysis (Thompson, 1986); this is the first work that strongly solved Ostle. We determined that the initial position is a draw. We found positions that take 147 plies to win, assuming that both players always choose the best move, and confirmed that 147 plies are the longest of all positions. Additionally, we performed a breadth-first search and proved that all positions targeted in the retrograde analysis are reachable from the initial position.

Retrograde analysis requires enumerating all possible positions of the game. Therefore, memory-saving techniques are crucial to solve larger games. In the ‘Methods’ section of this study, we provide an in-depth description about the techniques employed when applying retrograde analysis to solve Ostle.

**Figure 1:**
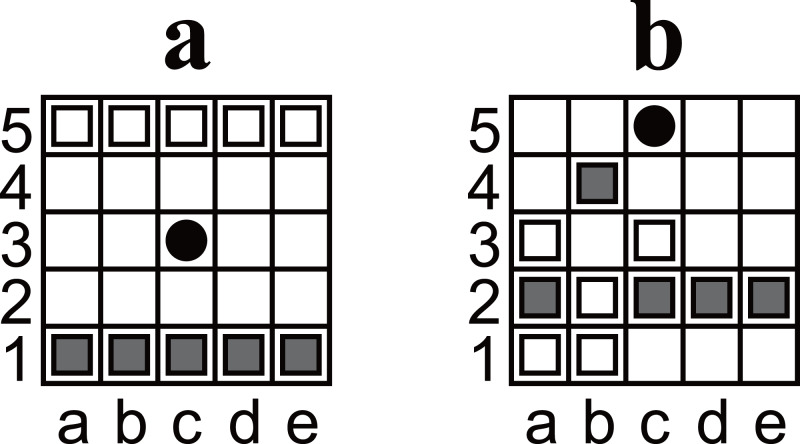
(A) An illustration of the initial position of Ostle. (B) Another example of a position. The position was discovered as a puzzle; if Black is the player to move, there is only one move that leads Black to win in seven plies. The answer is shown in Fig. 6.

In addition, we exhaustively enumerated positions in which sacrifice (*i.e.*, a move that voluntarily loses a piece) is necessary to win (*e.g.*, Fig. 1B). The significance of this work is not only that we have discovered interesting positions but also that we obtained helpful information to compose problems involving a tactical factor. For an experimental demonstration, we manually composed an Ostle problem with this information.

## Methods

### The rules of Ostle

Ostle is a two-player game. The players are referred to as Black and White. The initial position of Ostle is shown in Fig. 1A. The five dark gray pieces on the first rank are Black’s pieces; the white pieces on the fifth rank are White’s. The black circle on c3 is a “hole”. Note that ranks (rows) and files (columns) are not labeled with numbers and letters in the official explanation; the labels were added to enable chess-like algebraic notation.

**Figure 2:**
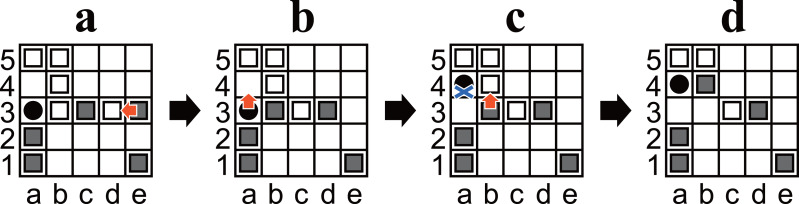
(A–D) Example diagrams of Ostle. Red arrows represent a chosen move. A blue cross mark represents a restriction.

The rules of Ostle are as follows:

1. Black moves first, after which the players alternate.
2. On each player’s turn, that player must choose either one of his/her owned pieces or the hole to be moved and move it in one square up, down, left, or right.
3. A pass is not allowed; both players must move.
4. If a piece reaches the hole or outside the board, it is removed from the game. Moving a player’s piece to the hole or outside the board is allowed.
5. Pieces can be moved to a square occupied by another piece. In this case, the original piece is pushed out and moved one square in the same direction. This process is recursive until a piece reaches an empty square, the hole, or the outside of the board (cf. Figs. 2A and 2C).
6. A player wins when the opponent has only three pieces left. For example, Black wins in Fig. 2D.
7. The holes can only be moved to empty squares. For example, in Fig. 2B, White moves the hole from a3 to a2 but cannot move it to b3 or a4 (because they are not empty) or to the left (because the left is outside the board).
8. Any position must not be the same as two plies before it. Any move that causes such a situation is restricted. For example, in Fig. 2C, Black cannot move the hole to a3 because the position after the ply would be exactly the same as the previous position (Fig. 2B).

Rule 8 inhibits one kind of repetition, but some repetitions are still possible. Therefore, Ostle, defined by the above rules, is not a finite game. In the following, we treat every repetition as a draw.

### Notation and preliminary

A formal model and useful notation are desribed below. We used the following components:

• A finite set *P* of **positions**, such that every *p* ∈ *P* corresponds to a unique arrangement of pieces on the board of an unfinished game, including the hole, as well as which player’s turn it is. Note that every *p* ∈ *P* includes either four or five white and four or five black pieces.
• A finite set *M*_*p*_ of **moves** associated to every *p* ∈ *P*. Moves are represented by an alphanumeric coordinates for the square and an uppercase letter for the direction (U, D, L, or R for up, down, left, or right, respectively). For example, the chosen moves in Fig. 2 are represented as e3L (Fig. 2A), a3U (Fig. 2B), and b3U (Fig. 2C).
• A finite set *S* of **states**, such that each *s* ∈ *S* consists of a unique tuple $({p}_{s},{m}_{s}^{r})$, where *p*_*s*_ ∈ *P* and ${m}_{s}^{r}\in ({M}_{{p}_{s}}\cup \{ \phi \} )$. The first element of the tuple identifies a position, and the second identifies which move is restricted (if applicable).
• A function *f*_*transition*_ that takes a position *p* ∈ *P* and its move *m* ∈ *M*_*p*_ as arguments, such that *f*_*transition*_(*p*, *m*) returns a state *s* ∈ *S* such that *p* transitions to *s* by *m*. A ply is represented by *f*_*transition*_.
• A function *f*_*square*_ associated with every *p* ∈ *P* takes a move *m* ∈ *M*_*p*_ as arguments, such that *f*_*square*_(*m*) returns *m*’s source square. For example, in Fig. 2A, if *m* is e3L, then *f*_*square*_e3L =e3.
• A function *f*_*direction*_ associated with every *p* ∈ *P* that takes a move *m* ∈ *M*_*p*_ as argument, such that *f*_*direction*_(*m*) returns the direction of *m*. For example, in Fig. 2A, if *m* is e3L, then *f*_*direction*_e3L =L.
• A boolean-valued function *f*_*if*_*PRS*_ takes a state *s* ∈ *S* as an argument, such that *f*_*if*_*PRS*_(*s*) returns True if and only if a position *p* ∈ *P* and an associated move *m* ∈ *M*_*p*_ exist such that *p* transitions to *s* by *m*. A **possibly reachable state** is a state *s* for which *f*_*if*_*PRS*_(*s*) is True. Note that it does not consider whether (*p*′, *m*) ∈ *S* or not. In other words, if *s* is not a possibly reachable state, *s* is guaranteed to be unreachable from any state; however, if *s* is a possibly reachable state, it does not follow that *s* is guaranteed to be reachable.
• A boolean-valued function *g* that takes a state *s* ∈ *S* as an argument, such that *g*(*s*) returns True if and only if a legal move ${m}^{{^{\prime}}}\in {M}_{{p}_{s}}\setminus \{ {m}_{s}^{r}\} $ exists such that *m*′ wins the game for the player to move. A **checkmate state** is a state *s* for which *g*(*s*) is True. For example, Fig. 2C is a checkmate state because Black wins with b3U. Additionally, a position *p* ∈ *P* is called a **checkmate position** if and only if all corresponding states of {*s* ∈ *S*:*p*_*s*_ = *p*∧*f*_*if*_*PRS*_(*s*) = *True*} are checkmate states.
• A boolean-valued function *h* that takes a state *s* ∈ *S* as an argument, such that *h*(*s*) = *f*_*if*_*PRS*_(*s*)∧(¬*g*(*s*)). A state *s* ∈ *S* is called a **non-trivial state** if and only if *h*(*s*) is True.
• Assume that *i*, *j*, *k* are integers and *i* ≤ *j* < *k*. Here, a bracket notation [*i*, *j*] indicates the integer interval between *i* and *j*, including both. Another bracket notation [*i*, *k*) also indicates an integer interval, but *k* is excluded. In other words, [*i*, *k*) = {*i*, *i* + 1, …, *k* − 1}.

Below are several theorems regarding the relationship between a checkmate state and checkmate position.

Theorem 1 *Any move which removes a piece cannot be restricted.*

Proof 1 *In Ostle, the number of pieces on the board decreases monotonically because no ply increases pieces. For this reason, for an arbitrary move *m* which removes a piece, the position after *m* is different from the position two plies before *m* in terms of the number of pieces. Therefore, *m* is never restricted by rule 8.* □

Theorem 2 *Any move which wins the game cannot be restricted.*

Proof 2 *Assume that a move *m* ∈ *M*_*p*_ from a position *p* ∈ *P* wins the game. This means that there are just four opponent’s pieces in *p*, and *m* removes an opponent’s piece. By the Theorem 1, we can conclude that *m* is never restricted.* □

Theorem 3 *If a state s* ∈ *S is a checkmate state, then the corresponding position p*_*s*_* is always a checkmate position.*

Proof 3 *The proof is by contradiction. Assume that there exists a state *s*′ ∈ *S* such that *s*′ is a checkmate state, but corresponding position *p*_*s*′_ is not a checkmate position. Then there must exist a move *m* ∈ *M*_*p*_*s*′__ such that *m* wins for the player to move.*

*From the definition of a checkmate position, there must exist a state *s*^∗^ ∈ *S* such that *p*_*s*^∗^_ = *p*∧*f*_*if*_*PRS*_(*s*^∗^) = *True*. Note that *m* ∈ *M*_*p*_*s*^∗^__ because a set of moves is associated only with a position, not a state. In order to satisfy the condition *f*_*if*_*PRS*_(*s*^∗^) = *True*, *m* must be restricted in the state *s*^∗^. This is in contradiction to Theorem 2.* □

### Move generation preliminaries

The details of a move generation algorithm are described below in Algorithm 1. Algorithm 1 generates all moves of an argument position in a predetermined order.


 
____________________________ 
Algorithm 1 G(p): Generate all moves in a predetermined order.__________________________________________ 
Require: p: A position. Note that the pieces must be labeled not as Black or White, but as Self 
     (the player to move) or Opponent. 
  1:  a ← an empty list 
  2:  b ←{‘a’,‘b’,‘c’,‘d’,‘e’}×{‘1’,‘2’,‘3’,‘4’,‘5’} 
 3:  b ← sort(list(b))                                ⊳ A list of all squares’ names in lexicographical order. 
  4:  for s ∈ b do 
 5:       if The hole exists on square s then 
 6:            if The hole can be legally moved up then 
 7:                  a.append(s+‘U’) 
  8:            end if 
 9:            if The hole can be legally moved down then 
10:                  a.append(s+‘D’) 
11:            end if 
12:            if The hole can be legally moved left then 
13:                  a.append(s+‘L’) 
14:            end if 
15:            if The hole can be legally moved right then 
16:                  a.append(s+‘R’) 
17:            end if 
18:       else if A Self’s piece exists on square s then 
19:            for d ∈{‘U’,‘D’,‘L’,‘R’} do 
20:                  a.append(s + d) 
21:            end for 
22:       end if 
23:  end for 
24:  return a_______________________________________________________________________________________________    


Note that Algorithm 1 does not consider the restriction of rule 8 and generates a restricted move for computational efficiency. In other words, Algorithm 1 is a “pseudo-legal” move generator; it is guaranteed that Algorithm 1 generates all legal moves, but each generated move is not guaranteed to be legal.

In addition, note that a position *p* ∈ *P* and multiple moves *m*_1_, …, *m*_*n*_ ∈ *G*(*p*) (2 ≤ *n* ≤ 4) exist such that for all *i* ∈ [1, *n*], *m*_*i*_ transitions *p* into the same position *p*′ ∈ *P*. For example, in the initial position, the three moves “a1D”, “a1L”, and “a1R” brings the same position (the piece on a1 is removed and everything else remains the same).

However, for an arbitrary position *p* ∈ *P*, the number of restricted moves in the return value of *G*(*p*) is at most one. In order to show this, there are several theorems in the following.

Theorem 4 *For all p* ∈ *P and m*_1_, *m*_2_ ∈ *M*_*p*_*, if f*_*square*_(*m*_1_) ≠ *f*_*square*_(*m*_2_)*, then f*_*transition*_(*p*, *m*_1_) ≠ *f*_*transition*_(*p*, *m*_2_)*.*

Proof 4 *After an arbitrary move, the square from which the piece was moved becomes empty. In contrast, the other squares never become empty if they were originally not empty. Therefore, *f*_*transition*_(*p*, *m*_1_) ≠ *f*_*transition*_(*p*, *m*_2_) in terms of whether the square from which the piece was moved is empty.* □

Theorem 5 *For all p* ∈ *P and m*_1_, *m*_2_ ∈ *M*_*p*_* ( m*_1_ ≠ *m*_2_*), if neither m*_1_* nor m*_2_* remove any pieces, then f*_*transition*_(*p*, *m*_1_) ≠ *f*_*transition*_(*p*, *m*_2_)*.*

Proof 5 *According to theorem 4, if *f*_*square*_(*m*_1_) ≠ *f*_*square*_(*m*_2_), then *f*_*transition*_(*p*, *m*_1_) ≠ *f*_*transition*_(*p*, *m*_2_). In the following, we will consider the case where *f*_*square*_(*m*_1_) = *f*_*square*_(*m*_2_). Let us denote *Q* = *f*_*square*_(*m*_1_) = *f*_*square*_(*m*_2_). Because *m*_1_ ≠ *m*_2_, *m*_1_ and *m*_2_ differ in the directions. Because neither *m*_1_ nor *m*_2_ remove any pieces, for each *i* ∈ {1, 2}, there is one square that is empty at *p* but filled at *f*_*transition*_(*p*, *m*_*i*_); let us denote the square by *Q*_*i*_. Note that *Q*_*i*_ is in the direction of *m*_*i*_ from *Q*. Then *Q*_1_ ≠ *Q*_2_ if *m*_1_ ≠ *m*_2_, and *m*_1_ and *m*_2_ are in different directions. Consequently, if *m*_1_ ≠ *m*_2_ and *f*_*square*_(*m*_1_) = *f*_*square*_(*m*_2_), then *f*_*transition*_(*p*, *m*_1_) ≠ *f*_*transition*_(*p*, *m*_2_) in terms of the square that is empty at *p* but filled at *f*_*transition*_(*p*, *m*_*i*_).* □

Theorem 6 *For all p* ∈ *P, the number of restricted moves in G*(*p*)* is at most one.*

Proof 6 *Taking the contraposition of theorem 5, we can find that for all *m*_1_, *m*_2_ ∈ *G*(*p*) (*m*_1_ ≠ *m*_2_)), if *f*_*transition*_(*p*, *m*_1_) = *f*_*transition*_(*p*, *m*_2_), then *m*_1_ and *m*_2_ are moves removing a piece. Using theorem 1, we can find that such *m*_1_ and *m*_2_ are never restricted. Taking the contraposition of this, we can conclude that for all *m*, *m*′ ∈ *G*(*p*) (*m* ≠ *m*′), if *m* is restricted, then *f*_*transition*_(*p*, *m*) ≠ *f*_*transition*_(*p*, *m*′), hence *f*_*transition*_(*p*, *m*′) is not restricted. Therefore, for all *p* ∈ *P* and *m* ∈ *G*(*p*), if *m* is restricted, then all the other moves in *G*(*p*) never restricted.* □

The return value of Algorithm 1 is a list of moves. It is essential for further analysis that the order, as well as the members, is deterministic, because in further analysis, every state in an arbitrary position is assigned a unique serial number based on the index of the restricted move in the list. This numbering method works correctly only if at most one move is restricted. Although Algorithm 1 is a pseudo-legal move generator, the number of restricted moves is guaranteed to be at most one by Theorem 6.

### Positional symmetry

Symmetry inherent in Ostle can make further analysis, including retrograde analysis, more efficient without losing any essential information. Specifically, each position has at most eight symmetric positions, including itself. Algorithm 2 enumerates these positions.


 
_____________________________________________________________________________________________________________ 
Algorithm 2 E(p): Enumerate all symmetric positions._______________________________________________________ 
Require: p: A position (e.g., a 5 × 5 matrix). 
Require: flip_lr(p): A function that horizontally flips the argument position. 
Require: flip_ud(p): A function that vertically flips the argument position. 
Require: transpose(p): A function that transposes the argument position. 
  1:  a ← an empty set 
  2:  for i ← [0,7] do 
 3:       x ← p 
 4:       if (i&1) ⁄= 0 then                    ⊳ The “&” symbols refer to the bitwise-and operation. 
  5:            x ← flip_lr(x) 
  6:       end if 
 7:       if (i&2) ⁄= 0 then 
 8:            x ← flip_ud(x) 
  9:       end if 
10:       if (i&4) ⁄= 0 then 
11:            x ← transpose(x) 
12:       end if 
13:       a.add(x) 
14:  end for 
15:  return a_______________________________________________________________________________________________    


In the following analysis, it was often helpful to consider symmetric positions as identical. Algorithm 3 was used to obtain a unique representative position among the symmetric positions.

 
_____________________________________________________________________________________________________________ 
Algorithm 3 U(p): Get a unique representative position among the symmetric positions.__________ 
Require: p: A position (e.g., a 5 × 5 matrix). 
Require: ptoi(p): An injective function that maps positions to integers. 
  1:  a ← p 
 2:  for x ∈ E(p) do 
 3:       if ptoi(x) < ptoi(a) then 
 4:            a ← x 
 5:       end if 
 6:  end for 
 7:  return a_______________________________________________________________________________________________    

Algorithm 3 calls a “ptoi” function that injectively maps positions into integers. Any mapping is acceptable as long as it is injective. In our implementation for this study, we represented a position itself to be a 55-bit integer. Two bitboards of pieces needed 25 bits each, and the remaining five bits were for the square of the hole. Therefore, the “ptoi” function was not employed (in other words, it was an identity map).

### Enumerating positions

Before the analysis, the possible positions of Ostle were exhaustively enumerated. Enumeration was based on the following criteria.

**Figure 3:**
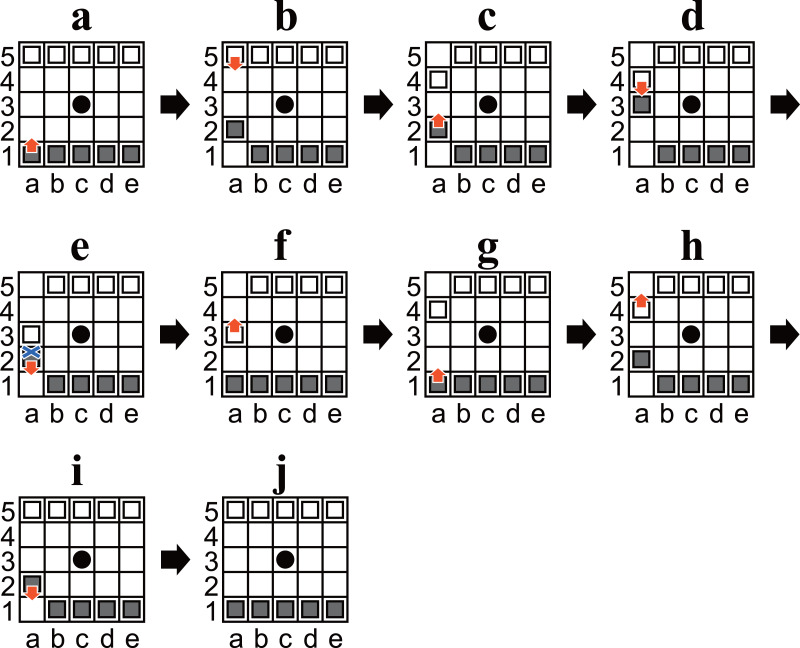
(A–J) Diagrams of Ostle illustrating a sequence to change the player to move in nine plies. A and J are identical, except for the player to move; (A) is Black’s turn, but (J) is White’s turn.

**Figure 4:**
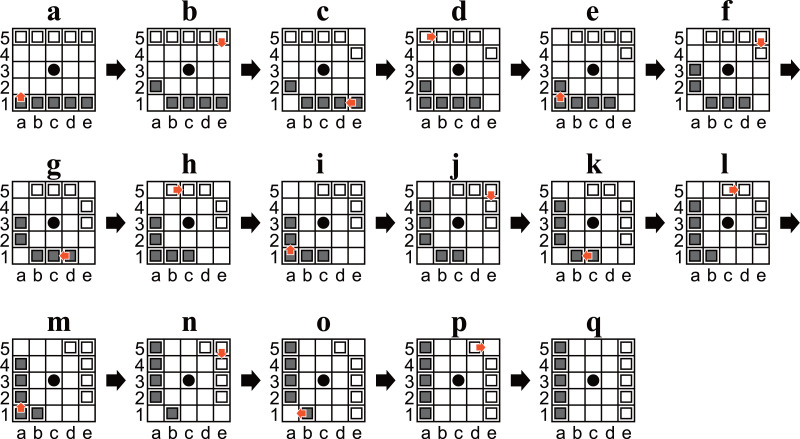
(A–Q) Diagrams of Ostle illustrating a sequence to rotate the initial position ninety degrees in sixteen plies.

• Checkmate positions were included in the enumeration. In contrast, positions after the game were over (*i.e.*, positions where a loser had only three pieces) were not included.
• Both players had no obligation to win in any checkmate position. In other words, positions that are unreachable from the initial position without overlooking a winning move were included in the enumeration.
• Only positions in which it was Black’s turn to move were enumerated. In other words, in the following, “Black” means “the player to move”, and “White” means “their opponent”, except where specifically noted otherwise. This is sufficient because there is a sequence of moves whereby the same position is reached, but the player to move is changed (an example is shown in Fig. 3).
• Symmetric positions were considered identical. This is sufficient because there is a sequence of moves to rotate the initial position ninety degrees (an example is shown in Fig. 4). The other symmetric positions can be reached by repeating the sequence two or three times.

The two observations below enable us to reduce the number of enumerating positions without losing exhaustiveness.

#### Observation 1

Let us consider the procedure of choosing the place of the hole first and then the pieces (white and black, five or four pieces each) in the remaining 24 squares. This procedure can construct an arbitrary position, but symmetric positions are enumerated separately. Here, if the symmetric positions are to be considered identical later, only six squares, a1, a2, a3, b2, b3, and c3, are sufficient to be considered for the hole placement. This is because if the hole is placed on one of the remaining 19 squares and the pieces on arbitrary squares, there always exist a symmetric position such that the hole is on one of the six squares.

#### Observation 2

There is never a symmetric relationship between two boards if their holes are on different squares of the above six squares. Moreover, there is never a symmetric relationship between two boards if the number of pieces of at least one player is different. Therefore, the positions could be divided into 24 cases according to the “place of the hole and number of pieces”. This categorization is mutually exclusive and collectively exhaustive. The detection of symmetric positions could be done by considering only the inside of each divided subset.

#### Algorithms

Algorithm 4 exhaustively enumerates and sorts all possible positions. In the description, each algebraic coordinate was assigned a number in lexicographical order; a1 was assigned 0, a2 was assigned 1, b1 was assigned 5, and so on.

The reason for sorting the list of positions was to perform a binary search to find the index of an arbitrary position. Therefore, any sorting criterion is acceptable as long as the comparison is fast.

 
_____________________________________________________________________________________________________________ 
Algorithm 4 Enumerate and sort all positions.__________________________________________________________________ 
  1:  v ← an empty list 
  2:  for (b,w,h) ∈{4,5}×{4,5}×{0,1,2,6,7,12} do              ⊳ This for-loop is parallelizable. 
  3:       w ← an empty list 
  4:       p ← an empty position (e.g., a dictionary) 
  5:       w ← D(w,p,0,b,w,h)                                               ⊳ The function D is Algorithm 5. 
  6:       for i ∈ [0,len(w)) do 
 7:            w[i] ← U(w[i])                                                    ⊳ The function U is Algorithm 3. 
  8:       end for 
 9:       w.uniquify()  ⊳ e.g., in C++, std::sort and std::unique are available; in Python, list(set(w)) 
     is. 
10:       v.concatenate(w)               ⊳ If executed in parallel, this line must be in a critical section. 
11:  end for 
12:  return sort(v)________________________________________________________________________________________________________    


 
_________________________________________________________________________________________________________________________________ 
Algorithm 5 D(v,p,n,b,w,h): An auxiliary function of depth-first search to enumerate positions. 
Require: v: A list of positions. 
Require: p: An in-process position (e.g., a dictionary). 
Require: n: An integer representing a considering square ( n ∈ [0,25] ). 
Require: b: An integer representing a number of remaining Black’s pieces ( b ∈ [0,5] ). 
Require: w: An integer representing a number of remaining White’s pieces ( w ∈ [0,5] ). 
Require: h: An integer representing the square where the hole exists ( h ∈{0,1,2,6,7,12} ). 
  1:  if n = 25 then 
 2:       v.append(p) 
  3:       return v 
 4:  end if 
 5:  if n = h then 
 6:       p[n] ← ”hole” 
  7:       return D(v,p,n + 1,b,w,h) 
  8:  end if 
 9:  if b > 0 then 
10:       q ← p 
11:       q[n] ← ”black” 
12:       v.concatenate(D(v,q,n + 1,b − 1,w,h)) 
13:  end if 
14:  if w > 0 then 
15:       q ← p 
16:       q[n] ← ”white” 
17:       v.concatenate(D(v,q,n + 1,b,w − 1,h)) 
18:  end if 
19:  v.concatenate(D(v,p,n + 1,b,w,h)) 
20:  return v_______________________________________________________________________________________________ 


### Enumerating non-trivial states

2,735,147,685 positions were enumerated by Algorithm 4 discussed in more detail in the ‘Result’ section). From Theorem 6, we can say that each position *p* ∈ *P* contains at most 25 states; one of them has no restricted move, and the others have one restricted move. Note that the number of moves generated by Algorithm 1 is at most 24. Therefore, the number of states is at most 68,378,692,125 (=2, 735, 147, 685 × 25).

In the following, we define a one-to-one correspondence between states and [0, |*P*| × 25). Specifically, if a state *s* has no restriction move (*s* = (*p*_*s*_, *ϕ*)), *s* is mapped to *j* × 25, where *P*[*j*] = *p*_*s*_. Otherwise ($s=({p}_{s},{m}_{s}^{r})$), *s* is mapped to *j* × 25 + *k* + 1, where *P*[*j*] = *p*_*s*_, *k* ∈ [0, |*G*(*p*_*s*_)|), and $G({p}_{s})[k]={m}_{s}^{r}$. In the following, we denote a state *s* as the *i*th state if and only if *s* is mapped to *i*.

We hypothesized that the number of non-trivial states is significantly smaller than 68,378,692,125. In order to confirm this, we developed Algorithm 6, which takes a sorted list of positions as an argument and returns a bitvector that represents whether each state is non-trivial. Note that the outermost for-loop of Algorithm 6 is parallelizable, but if parallelized, the operation of setting a bit of *v* must be atomic or executed in a critical section.

 
_____________________________________________________________________________________________________________ 
Algorithm 6 B(P,c =False): Make a bitvector that represents whether each state is non-trivial._ 
Require: P: A sorted list of all positions (return value of Algorithm 4). 
Require: c: A boolean flag to control whether checkmate positions are counted. 
  1:  v ← a zero-filled bitvector whose length is |P|× 25. 
  2:  for p ∈ P do ⊳ This for-loop is parallelizable; but if parallelized, the operation of setting a bit 
     of v must be atomic or executed in a critical section. 
  3:       M ← G(p)                                                      ⊳ Generate all moves of the position p. 
  4:       for m ∈ M do 
 5:            s ← ftransition(p,m) 
  6:            if c =True or g(s) =False then 
 7:                  i ← the integer such that P[i] = U(E(ps))              ⊳ e.g., perform a binary search. 
  8:                  M′ ← G(P[i])         ⊳ Generate all moves of the position P[i]. Note that |M′|≤ 24. 
  9:                  for j ∈ [0,|M′|) do 
10:                       s′ ← ftransition(P[i],M′[j]) 
11:                       if U(E(ps′)) = p then 
12:                            v[i × 25 + j + 1] ← 1                   ⊳ Set the (i × 25 + j + 1)-th bit of v to 1. 
13:                            goto END: 
14:                       end if 
15:                  end for 
16:                  v[i × 25] ← 1                                                 ⊳ Set the (i × 25)-th bit of v to 1. 
17:                  END: 
18:            end if 
19:       end for 
20:  end for 
21:  return v_______________________________________________________________________________________________    

### Bitvector and succinct indexable dictionary

Algorithm 6 eturns a bitvector *v*, whose length is 68,378,692,125 (It equals |*P*| × 25). Let us denote the *i*th bit of *v* as *v*[*i*]. For all *i* ∈ [0, |*v*|), it is guaranteed that *v*[*i*] = 1 if and only if the *i*th state is non-trivial. Consequently, 11,148,725,918 states were non-trivial (discussed in more detail in the ‘Result’ section).

For retrograde analysis, an array must be allocated to record the theoretical values of game states. If a 16-bit integer is allocated for each state, it will consume more than 136 GB of RAM. However, if allocated only for non-trivial states, memory consumption would be reduced to less than 23 GB of RAM. Let us denote the array only for non-trivial states as *x*.

To access the *i*th state on *v*, it is necessary to find its index number on *x*. The index equals the number of bits standing in the range [0, *i*) on *v*. It can be obtained by a query called a “rank query to a bitvector”, which returns the number of bits standing from the top to the *i*th bit in the bitvector.

Under the assumption that the bitvector is unchanged after initialization, it is known that the rank query can be processed in constant time by providing an auxiliary data structure called “succinct indexable dictionary” (Jacobson, 1988). It is also known to have the advantage that the space complexity of “succinct indexable dictionary” can be reduced to *o*(|*v*|). Nevertheless, since we only consider solving Ostle in the present study, it is unnecessary to focus on computational complexity theory (as it is the asymptotic behavior when |*v*| goes to infinity). In our implementation, the additional size of the dictionary for *v* was $ \frac{257}{1024} $ of the size of *v* itself.

Let us denote a bitvector with its succinct indexable dictionary as a “succinct bitvector”.

### Retrograde analysis

For all non-trivial states, retrograde analysis was performed to obtain the theoretical value of the state. We determined whether each state was a win, a loss, or a draw for the player to move. We also determined the number of plies required to reach a checkmate state, assuming that the winner is minimizing and the loser is maximizing it.

In a naive implementation of retrograde analysis, a game graph (*i.e.*, a directed graph with the states as nodes and the moves as edges) is initially constructed. However, in this study, we implemented Algorithm 7, which performs retrograde analysis of Ostle without explicitly having a game graph.

Algorithm 7 returns a vector of integers; each integer represents the theoretical value of a corresponding state. If it is zero, the state is a draw. If it is a negative number, the state is a loss for the player to move. If it is a positive number, the state is a win for the player to move.

 
_____________________________________________________________________________________________________________ 
Algorithm 7 Retrograde analysis of Ostle________________________________________________________________________ 
Require: A(i,v,x,y,lwin,llose,ldraw): Auxiliary function, which is described below as Algorithm 
     8. 
  1:  P ← A sorted list of all positions (i.e., return value of Algorithm 4). 
  2:  v ← B(P)                                                                                ⊳ B(P) is Algorithm 6. 
  3:  Convert v into succinct bitvector which supports rank query in constant time. 
  4:  x ← A zero-filled vector of which length is popcount(v) = 11,148,725,918. 
  5:  while True do 
 6:       y ← x 
 7:       for i ∈ [0,|P|) do                                               ⊳ This for-loop is parallelizable. 
  8:            lwin,llose,ldraw ← empty lists. 
  9:            M ← G(P[i])                                            ⊳ generate all moves of the position P[i]. 
10:            for j ∈ [0,|M|) do 
11:                  s ← ftransition(P[i],M[j]) 
12:                  k ← the integer such that P[k] = U(E(ps))                   ⊳ perform a binary search. 
13:                  if g(s) then                                                      ⊳ s is a checkmate state. 
14:                       llose.append((−1,j)) 
15:                  else 
16:                       if r ∈ [0,24) exists such that G(P[k])[r] is a restricted move then 
17:                            k ← k + r + 1 
18:                       end if 
19:                       if x[v.rank(k)] is a negative number then 
20:                            lwin.append((−x[v.rank(k)] + 1,j)) 
21:                       else if x[v.rank(k)] is a positive number then 
22:                            llose.append((−x[v.rank(k)] − 1,j)) 
23:                       else 
24:                            ldraw.append(j) 
25:                       end if 
26:                  end if 
27:            end for 
28:            Sort the elements of lwin and ones of llose in ascending order. 
29:            y ← A(i,v,x,y,lwin,llose,ldraw)  ⊳ If parallelized, this line must be in a critical section. 
30:       end for 
31:       if y = x then                                     ⊳ i.e., no state was updated in this iteration. 
32:            break 
33:       end if 
34:       x ← y 
35:  end while 
36:  return x_______________________________________________________________________________________________    


 
_____________________________________________________________________________________________________________ 
Algorithm 8 A(i,v,x,y,lwin,llose,ldraw): Auxiliary function for retrograde analysis of Ostle______ 
Require: i,v,x,y,lwin,llose,ldraw: variables appear in Algorithm 7. 
Require: Elements of lwin and ones of llose is already sorted in ascending order. 
  1:  λ(n) = if n ≤ 0, return inf; otherwise, return n.                ⊳ A function used in the following. 
  2:  λternary(a,b,c) = if a is True, return b; otherwise, return c. ⊳ A function used in the following. 
  3:  for j ∈ [0,25) do 
 4:       if v[i× 25 + j] = 1 then ⊳ Only if the best move is restricted, choose the second-best move. 
  5:            k ← v.rank(i × 25 + j) 
  6:            if lwin has two or more elements then 
 7:                  y[k] ← λternary(j = 1 + lwin[0][1],min(λ(x[k]),lwin[1][0]),min(λ(x[k]),lwin[0][0])) 
  8:            else if lwin has only one element, and ldraw has one or more elements then 
 9:                  y[k] ← λternary(j = 1 + lwin[0][1],max(x[k],0),min(λ(x[k]),lwin[0][0])) 
10:            else if lwin has only one element, and ldraw has no element then 
11:                  y[k] ← λternary(j = 1 + lwin[0][1],max(x[k],llose[0][0]),min(λ(x[k]),lwin[0][0])) 
12:            else if lwin has no element, and ldraw has two or more elements then 
13:                  y[k] ← max(x[k],0) 
14:            else if lwin has no element, and ldraw has only one element then 
15:                  y[k] ← λternary(j = 1 + ldraw[0],max(x[k],llose[0][0]),max(x[k],0)) 
16:            else if Neither lwin nor ldraw has any element then 
17:                  y[k] ← λternary(j = 1 + llose[0][1],max(x[k],llose[1][0]),max(x[k],llose[0][0]) 
18:            else 
19:                  assert False 
20:            end if 
21:       end if 
22:  end for 
23:  return y_______________________________________________________________________________________________ 


### Breadth-first search to prove the reachability

In order to prove that all possibly reachable states are reachable from the initial state, a breadth-first search was performed. Algorithm 7 represents the breadth-first search. The initial position is assumed to be the starting point (distance is zero), and the distance is assumed to be increased by one for each transition. We calculated the minimum distance of all possibly reachable states. Consequently, it was confirmed that all possibly reachable states are reachable from the initial position by finite plies.


 
_____________________________________________________________________________________________________________ 
Algorithm 9 Breadth-first search of Ostle________________________________________________________________________ 
  1:  P ← A sorted list of all positions (i.e., return value of Algorithm 4). 
  2:  v ← B(P, True)                                                                         ⊳ B(P) is Algorithm 6. 
  3:  Convert v into succinct bitvector which supports rank query in constant time. 
  4:  x ← A inf-filled vector of which length is popcount(v). 
  5:  I ← The index such that P[I] is the initial position shown in Fig. 1A. 
  6:  x[v.rank(I × 25)] ← 0 
  7:  for d ∈ [0,inf) do 
 8:       y ← x 
 9:       for i ∈ [0,|P|) do                                               ⊳ This for-loop is parallelizable. 
10:            M ← G(P[i])                                            ⊳ generate all moves of the position P[i]. 
11:            for j ∈ [0,25) do 
12:                  if x[v.rank(i × 25 + j)] = d then 
13:                       M′ ← G(P[i])                                  ⊳ generate all moves of the position P[i]. 
14:                       for k ∈ [0,|M′|) ∖{j − 1} do 
15:                            s ← ftransition(P[i],M[k]) 
16:                            l ← the integer such that P[l] = U(E(ps))     ⊳ e.g., perform a binary search. 
17:                            a ← v.rank(l × 25 + k) 
18:                            y[a] ← min(d + 1,x[a]) ⊳ If parallelized, this line must be in a critical section. 
19:                       end for 
20:                  end if 
21:            end for 
22:       end for 
23:       if y = x then                                     ⊳ i.e., no state was updated in this iteration. 
24:            break 
25:       end if 
26:       x ← y 
27:  end for 
28:  return x_______________________________________________________________________________________________    


### Computational resource

A c5.9xlarge instance of Amazon EC2 was used for all analyses performed in this study. The specifications were Intel Xeon Platinum 8124M CPU @ 3.00 GHz, 18 physical cores, two threads per core, and 72 GB of RAM.

We also executed the same analyses on a PC, of which specifications were AMD Ryzen 5950X CPU @ 3.40 GHz, 16 physical cores, two threads per core, and 128 GB of RAM. We verified that the results were the same as the ones obtained by the former analyses.

## Results

### Enumerating positions

**Figure 5:**
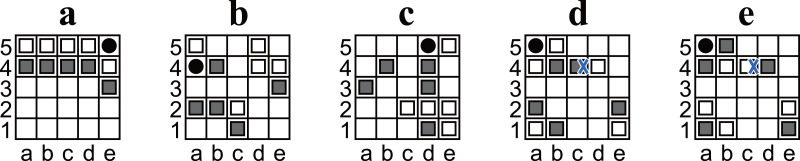
Diagrams of Ostle illustrating special positions and states. (A) An unreachable position if Black is the player to move. (B, C) States taking 147 plies to win (Black is the player to move). (D, E) States taking numerous plies to reach from the initial position (Black is the player to move in (D), and White is the player to move in (E)).

We firstly enumerated all possible positions by Algorithm 4. Table 1 shows the result. In total, 2,735,147,685 positions were obtained. In those enumerated positions, 399,102,582 were checkmate positions (14.5916%). Note that the enumerated positions included ones that were unreachable from the initial position (for example, the position in Fig. 5A is unreachable if Black is the player to move).

**Table 1:** The number of enumerated positions. C* means coordinate of the hole. P* is the number of pieces of the player to move. O* means ones of the opponent. N* is the number of enumerated positions.

C*	P*	O*	N*
a1	5	5	247,127,256
a2	5	5	494,236,512
a3	5	5	247,127,256
b2	5	5	247,127,256
b3	5	5	247,127,256
c3	5	5	61,788,564
a1	5	4	82,378,152
a2	5	4	164,745,504
a3	5	4	82,378,152
b2	5	4	82,378,152
b3	5	4	82,378,152
c3	5	4	20,598,588
a1	4	5	82,378,152
a2	4	5	164,745,504
a3	4	5	82,378,152
b2	4	5	82,378,152
b3	4	5	82,378,152
c3	4	5	20,598,588
a1	4	4	25,744,590
a2	4	4	51,482,970
a3	4	4	25,744,590
b2	4	4	25,744,590
b3	4	4	25,744,590
c3	4	4	6,438,855

P*	O*	N*
5	5	1,544,534,100
5	4	514,856,700
4	5	514,856,700
4	4	160,900,185

**Figure 6:**
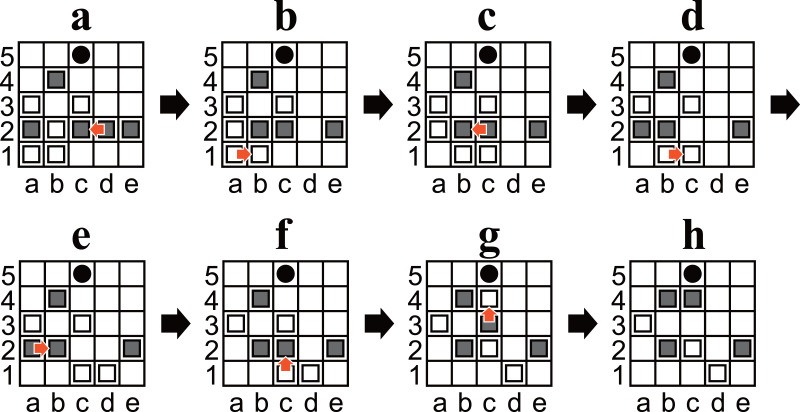
(A–H) Diagrams of Ostle illustrating a sequence in which that Black wins in seven plies. (A) is the same position as Fig. 1B.

### Obtaining non-trivial states

Because each position contains at most 25 states, the total number of states is at most 68,378,692,125 (=2, 735, 147, 685 × 25). However, the total number of non-trivial states could be much smaller. For the above reasons, we examined the total number of non-trivial states with Algorithm 6. Consequently, we confirmed that the number of non-trivial states was 11,148,725,918 (≈0.163 × 68, 378, 692, 125). As explained in the ‘Methods’ section, we reduced the memory usage of the following analysis by using this fact and the succinct indexable dictionary (Jacobson, 1988).

### Retrograde analysis

A retrograde analysis was performed, shown in Algorithm 7, to determine the theoretical values of all game states. The initial position is a draw. Table 2 shows the result. The summation of numbers of states in Table 2 equals the number of non-trivial states. Note that the “number of plies” in Table 2 is the number of plies to reach a checkmate state, so positions of an even number of plies are winning and those of an odd number are losing. The analysis took 35 h to compute using the computing environments described above. The file output of the result was 97.4 GB, a size that can be handled by modern inexpensive storage devices. (As declared in the Data Availability section below, the output files can be downloaded at figshare: https://doi.org/10.6084/m9.figshare.19668789.v1).

**Table 2:** Number of plies to reach any of the checkmate positions, and the number of such states. inf means draw. P* is the number of plies. N* is the number of such states.

P*	N*	P*	N*	P*	N*	P*	N*
inf	339,367,091	40	31,679,932	80	352,810	120	690
1	577,327,477	41	27,408,599	81	291,770	121	425
2	1,208,259,074	42	25,272,339	82	285,312	122	433
3	250,385,204	43	21,858,443	83	233,005	123	282
4	514,915,495	44	20,072,851	84	229,210	124	387
5	294,380,826	45	17,341,986	85	186,949	125	308
6	569,040,388	46	15,898,815	86	179,324	126	321
7	352,821,271	47	13,731,415	87	144,595	127	212
8	559,455,180	48	12,600,718	88	138,428	128	226
9	379,808,723	49	10,908,242	89	113,388	129	205
10	535,425,081	50	9,994,880	90	108,165	130	179
11	379,563,356	51	8,667,693	91	86,970	131	211
12	462,977,806	52	7,940,165	92	83,620	132	73
13	352,330,833	53	6,893,605	93	67,110	133	113
14	395,732,654	54	6,314,317	94	65,247	134	44
15	318,063,321	55	5,502,897	95	53,164	135	99
16	338,626,104	56	5,039,556	96	51,284	136	68
17	281,546,559	57	4,382,022	97	40,837	137	146
18	287,320,623	58	3,998,228	98	38,653	138	74
19	244,575,415	59	3,470,949	99	30,388	139	106
20	241,883,809	60	3,163,370	100	27,311	140	44
21	208,979,928	61	2,764,844	101	22,936	141	40
22	202,402,295	62	2,521,503	102	21,612	142	16
23	176,160,115	63	2,195,886	103	18,273	143	7
24	168,414,137	64	2,010,460	104	15,740	144	9
25	146,956,825	65	1,759,413	105	13,176	145	4
26	139,319,551	66	1,595,483	106	10,996	146	7
27	121,669,807	67	1,400,525	107	9,486		
28	114,485,394	68	1,267,104	108	7,740		
29	99,874,325	69	1,108,248	109	7,290		
30	93,577,296	70	1,006,232	110	5,481		
31	81,527,252	71	878,861	111	5,015		
32	76,116,163	72	801,288	112	3,885		
33	66,122,478	73	700,241	113	3,741		
34	61,530,140	74	649,879	114	2,885		
35	53,329,309	75	561,353	115	2,593		
36	49,530,287	76	528,175	116	1,785		
37	42,847,011	77	449,395	117	1,447		
38	39,660,828	78	434,621	118	1,005		
39	34,312,611	79	361,217	119	771		

### States taking 147 plies to win

Table 2 shows that there are seven states taking 147 plies to win, and 147 is the longest number. The seven states comprises two positions shown in Figs. 5B and 5C. The two positions with no restricted move are included in the seven states. The remaining five states consist of the two positions with some restricted move.

In detail, in the position shown in Fig. 5B, states where either c3U, b2R, e3U, or a4D is restricted takes 147 plies to win. On the other hand, a state where a2R is restricted is the state that ended in lose in a maximum of 58 plies. In the position shown in Fig. 5C, a state where d5L is restricted takes 147 plies to win.

### Breadth-first search

In order to prove that all possibly reachable states obtained are reachable from the initial position, a breadth-first search was performed (shown in Algorithm 9), which confirmed that all of them were reachable. Table 3 shows the results of the breadth-first search. The analysis took at most 6 h to compute using the computing environments described above. The file output of the result was 114 GB, a size that can be handled by modern inexpensive storage devices. (As declared in the Data Availability section below, the output files can be downloaded at figshare: https://doi.org/10.6084/m9.figshare.19668789.v1).

**Table 3:** Number of plies to reach states from the initial state, and the number of such states. P* is the number of plies. N* is the number of such states.

P*	N*	P*	N*
0	1	14	411,886,389
1	9	15	767,525,717
2	102	16	1,262,744,615
3	954	17	1,851,900,832
4	6,329	18	2,259,589,185
5	33,052	19	2,356,709,939
6	147,620	20	1,884,609,912
7	556,811	21	1,172,437,043
8	1,863,530	22	475,193,903
9	5,542,830	23	113,051,575
10	15,200,179	24	9,503,831
11	38,307,337	25	115,519
12	91,419,758	26	97
13	201,637,267		

Table 3 shows that there are 97 states that take 26 plies to reach from the initial position, and 26 is the largest number. For example, the state shown in Fig. 5D is one of the 97 states. Note that the algorithm identifies symmetric positions and which player is the player to move. Therefore, if they were not identified, a state would be found that takes 27 or more plies to reach from the initial position. However, all states identified by this algorithm are guaranteed to be reachable; by adding the sequences shown in Figs. 3 and 4 in the opening, we can say that all symmetric states (and states in which the player to move is changed) are also reachable. The above matters are explained in more detail below.

Algorithm 9 outputs that the state shown in Fig. 5D takes 26 plies to reach from the initial position. In the eight symmetric states, including the one shown in Fig. 5D, there exists a state that can be reached in 26 moves from the initial position, and 26 moves is the least for the eight positions. In other words, all the symmetric states are reachable in just 26 moves from the initial position or one of its symmetric positions. Figure 4 implies that all four positions are reachable. Consequently, we can say that all the eight symmetric states are reachable.

The state shown in Fig. 5E (where White is the player to move) was also treated as the same state as the one in Fig. 5D. By adding the sequence of moves shown in Fig. 3 in the opening, we can say that the state shown in Fig. 5E (where White is the player to move) is reachable from the initial position.

As a side note, every state in which White is the player to move takes an odd number of plies to reach from the initial position. Because the result shows that 26 plies is the least number needed to reach the identified states, The state shown in Fig. 5E (White is the player to move) must take at least 27 plies.

### Discovering interesting states and composing a tactical problem from one of them

To demonstrate the usefulness of the retrograde analysis, we discovered interesting states from which to explore the nature of games. For example, we discovered states that are wins for the player to move, but he/she must choose sacrificing a piece to win. We discovered 35,107 such states, and Fig. 1B is an example. Figure 1B can be interpreted as a composed problem with a suitable stipulation (“Win in seven plies.”). Notably, Among the 35,107 games, some were not appropriate as an aesthetic composed problem because there were multiple solution that could be won in seven moves. We manually extracted the state and wrote appropriate stipulation to compose the problem.

#### Solution to the puzzle in Fig. 1B

Solution to the puzzle in Fig. 1B is “d2L,a1R,c2L,b1R,b2R,c1U,c3U” as shown in Fig. 6. In the solution, White chose the moves that maximize the number of plies before losing, and Black chose ones that minimize the number of plies. At the first position (Fig. 6A), “d2L” and “e2L” are the only choices to win. “e2L” is also a sacrificing move but takes nine plies to win.

## Discussion and future works

This study is the first to strongly solve Ostle and determine the theoretical values of all game states. We discovered that the initial position is a draw. Additionally, we found that there exist states from which it takes 147 plies to win, and those that take at least 26 plies to reach from the initial position.

Based on the results of the performed analysis, we discovered states in which sacrificing is necessary to win. Because sacrificing is a highly tactical move, we can say that the significance of the discovery is rooted in the nature of games. There might exist other tactics apart from sacrificing and seeking them is one of the goal for future work.

To demonstrate the usefulness of strongly-solving games, we manually composed a tactical puzzle of Ostle, which consisted of a state discovered through the analysis and a suitable stipulation added by us. We want to emphasize that strongly solving is a promising tool for composing puzzles, especially for recent popular pure strategy board games.

This article presents an in-depth examination of the techniques employed when applying retrograde analysis to solve Ostle. Such information will be beneficial for readers in solving other games in the future. However, it is worth mentioning that we could solve Ostle through the utilization of a single PC and on-memory capabilities. Utilizing multi-node and storage devices with recent computers, though noteworthy, is outside the scope of this study.

Based on the output of our retrograde analysis, software can easily be made that instantly chooses the best move for an arbitrary state of Ostle. Because the initial position is a draw, such software never loses. However, it cannot win unless the opponent makes a mistake. Here, it will be a future challenge to create a program that can lure the human opponent into a state where he/she is likely to make a mistake.

We solved Ostle in this study, but in principle every pure strategy board game is solvable. The reason we cannot solve larger games (*i.e.*, reversi, chess, and go) is due to lack of computing power and algorithms. Since computing power continues to improve year by year, solving larger famous games using future computer must be grand challenges achieved in the future.

## Conclusions

Many pure strategy board games are still unsolved and have interesting but undiscovered aspects, but computing power is limited. Therefore, to solve a wider variety of games at a more detailed level, it is essential to use techniques to reduce memory consumption and computation time for each game. In this study, we considered various properties of the subject game (such as the symmetry of the positions) and utilized various techniques such as succinct data structure and bitboards. Consequently, the analysis of Ostle could be performed in an inexpensive computing environment. We hope that this article and the source code help with future research on solving, analyzing, and extracting interesting facts from various other pure strategy board games.

## Additional Information and Declarations

### Data Deposition

The following information was supplied regarding data availability: The source code is available at GitHub and Zenodo: https://github.com/eukaryo/ostle_solver
Hiroki Takizawa. (2023). eukaryo/ostle_solver: v1.0.2.0 (v1.0.2.0). Zenodo. https://doi.org/10.5281/zenodo.8242219
The outputs of analyses are available at figshare: Takizawa, Hiroki (2022). ostle_solver_output.tar.bz2. figshare. Dataset. https://doi.org/10.6084/m9.figshare.19668789.v1.
